# The Association Between Constipation and Lower Urinary Tract Symptoms in Parous Middle-Aged Women: A Prospective Cohort Study

**DOI:** 10.1089/jwh.2020.8624

**Published:** 2021-08-17

**Authors:** Nour Alhababi, Maria Christine Magnus, Marcus John Drake, Abigail Fraser, Carol Joinson

**Affiliations:** ^1^ Population Health Sciences, Bristol Medical School, University of Bristol, Bristol, United Kingdom.; ^2^ MRC Integrative Epidemiology Unit, University of Bristol, Bristol, United Kingdom.; ^3^ Centre for Fertility and Health, Norwegian Institute of Public Health, Oslo, Norway.; ^4^ Bristol Urological Institute, Bristol Medical School, University of Bristol, Bristol, United Kingdom.

**Keywords:** LUTS, constipation, urinary incontinence, women's health, ALSPAC

## Abstract

***Objective:*** To examine the prospective association between constipation and risk of developing lower urinary tract symptoms (LUTS) in parous middle-aged women.

***Materials and Methods:*** The study uses data from 3,729 women from the Avon Longitudinal Study of Parents and Children who provided self-reports of medication intake for constipation at two time points (Baseline): 2001–2003 and 2003–2005. Women with LUTS at baseline were excluded. After 10 years of follow-up, women provided self-reports of LUTS using an adapted version of the International Consultation on Incontinence Questionnaire on Female LUTS. LUTS were categorized according to International Continence Society definitions as stress urinary incontinence (UI), urgency UI, mixed UI, nocturia, increased daytime frequency, urgency, hesitancy, and intermittency. LUTS were considered present if symptoms were reported to occur at least “sometimes” for all subtypes, except for increased daytime frequency (≥9 times) and nocturia (≥2 times nightly).

***Results:*** At follow-up, the prevalence of any LUTS was 40%. Women (mean age 43.3 years, standard deviation 0.5), who took medication for constipation at either time point had increased risks of urgency (adjusted relative risks [RRs] = 1.35; 95% confidence interval [CI] 1.04–1.95) and hesitancy (adjusted RR = 1.72; 95% CI 1.04–3.01) compared with women who reported not using medication for constipation at either time point. The risk of urgency (adjusted RR = 1.94; 95% CI 1.15–3.29) and hesitancy (adjusted RR = 1.78; 95% CI 1.03–4.19) was greater for women who reported taking medication for constipation at both time points. There was no evidence that constipation was associated with stress UI, urgency UI, mixed UI, nocturia, increased daytime frequency, and intermittency.

***Conclusion:*** Constipation is prospectively associated with an increased risk of urgency and hesitancy among parous middle-aged women. If further research finds evidence that this association is causal, this implies that women should seek treatment to alleviate constipation to reduce their consequent risk of developing these LUTS.

## Introduction

The International Continence Society (ICS) groups lower urinary tract symptoms (LUTS) into storage, voiding, and postmicturition symptoms.^1^ Stress urinary incontinence (UI) is the most common subtype of LUTS (prevalence ranging 10%–39%), followed by mixed UI (prevalence ranging 7.5%–25%), and then urgency UI (prevalence ranging 1%–7%).^2^

LUTS in women have been linked to lower quality of life due to interference with daily living,^3,4^ and are associated with a substantial psychological and economic burden.^5^ Established risk factors for LUTS include parity, delivery mode, older age, obesity, and hysterectomy.^2,6,7^ It has also been suggested that constipation could increase the risk of LUTS in women.^8,9^ This is because the urinary and bowel tracts are interrelated structures and their common embryology, overlapping innervation, and anatomical proximity could mean that dysfunction in the bowel may affect the bladder.^8,9^ Constipation is common and is estimated to affect 12%–32% of middle-aged women.^9^ The National Institute for Health and Care Excellence guidelines state that constipation and LUTS often co-occur and recommend screening for constipation in women while assessing and treating LUTS.^10^ The temporal relationship between constipation and LUTS is, however, unclear because previous studies are mostly cross-sectional. Other limitations of conducted studies include modest sample sizes, limited adjustment for potentially important confounders, such as physical activity and hysterectomy,^11–15^ and recall bias when participants report constipation retrospectively.

It is important to examine whether constipation is prospectively associated with developing LUTS as this could have important implications for prevention of LUTS. The aim of our study is to examine the prospective association between constipation and risk of LUTS in a large cohort of parous middle-aged women with a 10-year follow-up period.

## Materials and Methods

Our study includes women participating in the Avon Longitudinal Study of Parents and Children (ALSPAC).^16,17^ ALSPAC is a prospective population-based birth cohort study, which recruited 14,541 pregnant women residing in the former Avon Health Authority in England, with an estimated date of delivery between April 1991 and December 1992. Detailed information can be found at the cohort website (www.bristol.ac.uk/alspac), including a fully searchable data dictionary **(**www.bristol.ac.uk/alspac/researchers/our-data/). Ethics approval for the study was obtained from the ALSPAC Ethics and Law Committee and the Local Research Ethics Committees. The relevant Ethics Committee or Institutional Review Board provided (or waived) approval.

**FIG. 1.**
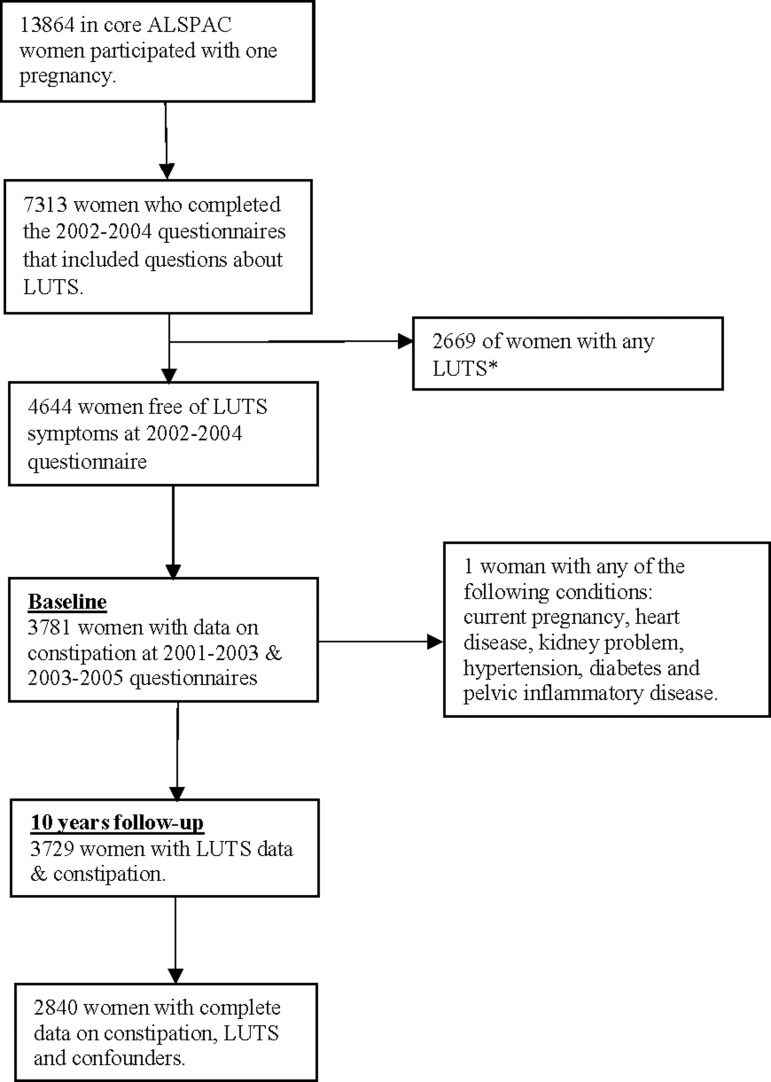
Sample flowchart for constipation and LUTS paper. *Any LUTS includes storage symptoms (stress UI, mixed UI, urgency UI, urgency, nocturia, increased daytime frequency) and voiding symptoms (hesitancy and intermittency). LUTS, lower urinary tract symptoms; UI, urinary incontinence.

We defined the baseline of the current study between 2001 and 2005, at which time we excluded prevalent cases of LUTS and classified women according to whether they were taking medication to treat constipation or not. A total of 3,922 women had information on constipation and had not experienced LUTS at baseline (Fig. 1). Of these women, 3,729 had information on LUTS after 10 years of follow-up and were included in the current study (2011–2012) (Fig. 1).

### Constipation

Use of medication for constipation was self-reported in questionnaires at two time points (between 2001–2003 and 2003–2005). Women were asked to indicate whether they had “used any medicines (pills, syrups, inhalers, drops, sprays, suppositories, pessaries, ointments *etc.*, including homeopathic and herbal remedies) in the last 12 months for constipation.” Women indicated “yes” in response to this question by checking a box, whereas all women who did not respond “yes” were assumed to not have used any medicines for constipation in the past 12 months. We categorized the responses to using medications for constipation during the two time points as: none, yes at one of the two time points, and yes at both time points.

To increase statistical power, we repeated the analysis by collapsing the constipation variable into two categories instead of three: women who reported “no constipation” and women who reported “taking medication for constipation at any time point.”

### Lower urinary tract symptoms

We chose to look at both storage and voiding symptoms due to their burden and impact on women's quality of life.^5,18^ Women completed a range of questions on LUTS that are drawn from the validated Bristol Female LUTS (BFLUTS) questionnaire at baseline (2002–2004).^19^ Women who reported LUTS symptoms (including stress UI, mixed UI, urgency UI, urgency, nocturia, increased daytime frequency, hesitancy and intermittency) at baseline were excluded. After 10 years of follow-up, women completed the International Consultation on Incontinence Questionnaire on Female LUTS (ICIQ-FLUTS).^20^ The ICIQ-FLUTS modular questionnaire used after 10 years of follow-up was adapted from the BFLUTS questionnaire. It includes the same questions as the BFLUTS, in addition to questions capturing women's ratings of whether, and to what degree, they rated the LUTS as bothersome. Based on the ICS definition, we categorized the responses according to different types of LUTS, including stress UI, urgency UI, mixed UI, urgency, nocturia, increased daytime frequency, hesitancy, and intermittency. A high percentage of women (49% of women) reported waking up once at night to urinate compared with waking up twice at night (7% of women). Therefore, we additionally examined women's ratings of how bothersome they found waking up once a night to urinate versus twice a night. Waking once a night to urinate was generally not considered bothersome, therefore, we decided to define nocturia as waking up to urinate two or more times per night.^21^ We also examined any type of LUTS which includes any storage or voiding symptom.

Box 1 lists the LUTS related questions included in the BFLUTS and ICIQ-questionnaires, which were used to identify women with LUTS at baseline and at follow-up after 10 years. LUTS were considered present if symptoms were reported to occur at least “sometimes” for all subtypes, except for increased daytime frequency (≥9 times) and nocturia (≥2 times nightly). We were unable to include unexplained UI, enuresis, and straining to void because there were very small numbers of women reporting experiencing these LUTS, preventing any meaningful analysis. The LUTS questionnaires did not ask about other voiding symptoms, including slow stream, spraying of urinary stream, and terminal dribble.

### Other variables

Potential confounders that could influence both constipation and LUTS were identified by a detailed search of the literature and clinical knowledge. Confounders included age (self-reported at recruitment), educational qualifications categorized as university degree versus no degree (Certificate of Secondary Education, Vocational, Ordinary level, Advanced level), occupational social class (1991 British Office of Population and Census Statistics—dichotomized into manual and nonmanual), body mass index (BMI: calculated from self-reported height and weight at baseline), parity (self-reported by women at recruitment and categorized into 1, 2, 3, or more births), hysterectomy (self-reported by women at baseline and categorized into yes/no), and physical activity (self-reported at baseline and translated into total metabolic equivalents [MET] minutes per week and categorized into five categories: 0, 0.1–17.2, 17.3–29.2, 29.3–43.2, and >43.2 MET hours/week).

Characteristics of women may have changed during the follow-up period between exposure and outcome measures. Therefore, we re-ran the complete case analysis with updated variables, including age, BMI (measured at the time of outcome assessment), parity, and hysterectomy (measured 2 years before outcome assessment). We were unable to account for any potential changes in physical activity, education, or social class after baseline.

### Statistical analyses

We used log-binomial multivariable regression to estimate the association between constipation (none/at one time point/at both time points) and subtypes of LUTS, reporting relative risks (RRs) and 95% confidence intervals (CIs).

We used multiple imputation to impute missing covariate information for women considered to be eligible for the analysis (*i.e.*, women who provided data on constipation and LUTS). Missing information on all the confounders of interest was therefore imputed. We generated 20 datasets using multiple imputation by fully conditional specification (chained equations). The imputation model included constipation, all LUTS outcomes, and the confounders mentioned above. The main analysis results were obtained by averaging across the results from each of these 20 datasets using Rubin's rules. All analyses were done using STATA/MP 15 (StataCorp, TX).

## Results

Table 1 compares the distribution of characteristics in the complete-case and the multiple-imputed datasets for women included in analyses. The distribution of background characteristics was very similar before and after imputation. The most common type of LUTS was stress UI (23%). The prevalence of any LUTS at 10 years of follow-up was 40%.

**Table 1.** Distribution of Characteristics of Women Included in Analyses of Lower Urinary Tract Symptoms in Complete Case, Observed and Imputed Datasets

	Missing (*n*)	Observed data	Imputed data sets (%)* n* = 3,729
Age, mean (SE)	95	43.3 (0.08)	43.3 (0.01)
BMI, mean (SE)	369	24.3 (4.3)	24.3 (0.07)
Parity, *n* (%)	83		
1		546 (15)	559 (15)
2		1,776 (49)	1,827 (49)
3+		1,324 (36)	1,343 (36)
Hysterectomy, *n* (%)	95		
Yes		122 (3)	112 (3)
No		3,512 (97)	3,617 (97)
Occupational social class, *n* (%)	372		
Manual		564 (15)	634 (17)
Nonmanual		2,793 (85)	3,095 (83)
Physical activity (MET hours/week), *n* (%)	0		
0		472 (13)	485 (13)
0.1–17.2		733 (20)	709 (19)
17.3–29.2		813 (22)	820 (22)
29.3–43.2		907 (24)	895 (24)
≥43.2		804 (21)	820 (22)
University degree, *n* (%)	310		
Yes		261(8)	299 (8)
No		3,158 (92)	3,430 (92)
Constipation medication, *n* (%)	0		
None		3,415 (92)	3,431(92)
Only one time point		227 (6)	224 (6)
Both time points		87 (2)	74 (2)
Constipation medication, *n* (%)	0		
None		3,415 (92)	3,430 (92)
Any time point		314 (8)	299 (8)
Stress UI, *n* (%)	8		
Yes		844 (23)	858 (23)
No		2,877 (77)	2,871 (77)
Urgency UI, *n* (%)	13		
Yes		318 (9)	336 (9)
No		3,398 (91)	3,393 (91)
Mixed UI, *n* (%)	15		
Yes		304 (9)	299 (8)
No		3,410 (91)	3,430 (92)
Increased daytime frequency, *n* (%)	7		
Yes		531 (14)	552 (14)
No		3,191 (86)	3,207 (86)
Nocturia, *n* (%)	7		
Yes		330 (9)	336 (9)
No		3,392 (91)	3,393 (91)
Urgency, *n* (%)	6		
Yes		657 (18)	671 (18)
No		3,066 (82)	3,058 (82)
Any type of LUTS, *n* (%)	165		
Yes		1,443 (40)	1,492 (40)
No		2,121 (60)	2,237 (60)
Hesitancy, *n* (%)	52		
Yes		181 (6)	186 (5)
No		3,496 (94)	3,543 (95)
Intermittency, *n* (%)	15		
Yes		341 (9)	336 (9)
No		3,373 (91)	3,393 (91)

BMI, body mass index; SE, standard error; LUTS, lower urinary tract symptoms; UI, urinary incontinence; MET, metabolic equivalents.

**Box 1.** Box Lists the LUTS-Related Questions Included in the BFLUTS and ICIQ-Questionnaires, Which Were Used to Identify Women with LUTS at Baseline and at Follow-Up After Ten Years

LUTS subtypes	Definitions	Questions used to exclude women with LUTS 1 year after baseline (2002–2004)	Response option	Questions used as an outcome measure at 10 years of follow-up (2011–2012)	Response option
Stress UI	Complaint of involuntary leakage on effort or exertion, or on sneezing or coughing.^24^	In the past month, how often have you had any of the following: problem holding urine when you jump, sneeze *etc.*	**1**—**Almost all the time** **2**—**Sometimes** 3—Not at all	Does urine leak when you are physically active, exert yourself, cough, or sneeze?	1—Never 2—Occasionally **3**—**Sometimes** **4**—**Most of the time** **5**—**All of the time**
Urgency UI	Complaint of involuntary loss of urine associated with urgency.^24^	Does urine leak before you can get to the toilet? How often do you have to rush to the toilet to urinate?	1—Never 2—Ocassionally **3**—**Sometimes** **4**—**Most times** **5**—**Every time** and 1—Never 2—Occasionally **3**—**Sometimes** **4**—**More often than not** **5**—**Every time**	Does urine leak before you can get to the toilet? Do you have a sudden need to rush to the toilet to urinate?	1—Never 2—Occasionally **3**—**Sometimes** **4**—**Most of the time** **5**—**All of the time** and 1—Never 2—Occasionally **3**—**Sometimes** **4**—**Most of the time** **5**—**All of the time**
Mixed UI	Complaint of involuntary leakage associated with urgency and also with exertion, effort, sneezing, or coughing.^24^	Does urine leak before you can get to the toilet? How often do you have to rush to the toilet to urinate? In the past month, how often have you had any of the following: problem holding urine when you jump, sneeze *etc.*	1—Never 2—Ocassionally **3**—**Sometimes** **4**—**Most times** **5**—**Every time** and 1—Never 2—Occasionally **3**—**Sometimes** **4**—**Most of the time** **5**—**All of the time** AND **1**—**Almost all the time** **2**—**Sometimes** 3—Not at all	Does urine leak before you can get to the toilet? Do you have a sudden need to rush to the toilet to urinate? Does urine leak when you are physically active, exert yourself, cough, or sneeze?	1—Never 2—Occasionally **3**—**Sometimes** **4**—**Most of the time** **5**—**All of the time** and 1—Never 2—Occasionally **3**—**Sometimes** **4**—**Most of the time** **5**—**All of the time** and 1—Never 2—Occasionally **3**—**Sometimes** **4**—**Most of the time** **5**—**All of the time**
Nocturia	Waking to pass urine during the main sleep period.^21^	During the night, how many times do you have to get up to urinate, on average?	1—None 2—One **3**—**Two** **4**—**Three** **5**—**Four or more**	During the night, how many times do you have to get up to urinate, on average?	1—None 2—One **3**—**Two** **4**—**Three** **5**—**Four or more**
Urgency	Complaint of a sudden compelling desire to pass urine, which is difficult to defer.^24^	How often do you have to rush to the toilet to urinate?	1—Never 2—Occasionally **3**—**Sometimes** **4**—**More often than not** **5**—**Everytime**	Do you have a sudden need to rush to the toilet to urinate?	1—Never 2—Occasionally **3**—**Sometimes** **4**—**Most of the time** **5**—**All of the time**
IDF	Complaint that micturition occurs more frequently during waking hours than previously deemed normal.^24^	During the day, how many times do you urinate (pass water or have a wee) on average?	1–6 Times 7–8 Times **9–10 Times** **11–12 Times** **13 or more times**	During the day, how many times do you urinate (pass water or have a wee) on average?	1–6 Times 7–8 Times **9–10 Times** **11–12 Times** **13 or more times**
Hesitancy	Complaint of a delay in initiating micturition.	How often is there a delay before you can start to urinate?	1—Never 2—Occasionally **3**—**Sometimes** **4**—**Most times** **5**—**Every time**	Is there a delay before you can start to urinate?	1—Never 2—Occasionally **3**—**Sometimes** **4**—**Most of the time** **5**—**All of the time**
Intermittency	Intermittent stream (intermittency) is the term used when the individual describes urine flow, which stops and starts on one or more occasions, during micturition.	Do you stop and start more than once while you urinate without meaning to?	1—Never 2—Occasionally **3**—**Sometimes** **4**—**Most times** **5**—**Every time**	Do you stop and start more than once while you urinate?	1—Never 2—Occasionally **3**—**Sometimes** **4**—**Most of the time** **5**—**All of the time**

Answers marked as bold were considered positive when defining the types of UI.

Urgency UI and mixed UI were identified only if women have responded positively to all relevant questions

LUTS, lower urinary tract symptoms; BFLUTS, Bristol Female LUTS; ICIQ, International Consultation on Incontinence Questionnaire; IDF, increased daytime frequency; UI, urinary incontinence.

A total of 6% of women (*n* = 227) reported having used medications for constipation at one of the time points, whereas 2% of women (*n* = 87) used medications for constipation at both time points.

Table 2 shows women's characteristics according to use of medications for constipation. There was no strong evidence for differences in the proportion of women in the different constipation categories according to the confounders except for hysterectomy.

**Table 2.** Characteristics of Women According to Use of Medication for Constipation by Confounders

Confounder	Constipation medication			
	None at both time points (*n* = 3,415)	Only one time point (*n* = 227)	Both time points (*n* = 87)	*p*-Value
Age, years [mean (SD)]	43.3 (0.5)	43.2 (0.5)	43.3 (0.5)	0.125
BMI (kg/m^2^) [mean (SD)]	24.3 (4.3)	24.8 (4.2)	24.1 (3.9)	0.262
Hysterectomy, %				0.005
Yes	3	4	0	
Physical activity (MET hours/week), %				0.590
0	13	13	12	
0.1–17.2	20	21	15	
17.3–29.2	22	19	23	
29.3–43.2	24	25	26	
≥43.2	21	22	24	
Occupational social class, %				0.559
Manual	17	14	17	
Nonmanual	83	86	83	
University degree, %				0.932
Yes	8	8	9	
Parity, %				0.538
1	15	15	16	
2	49	52	47	
3+	36	33	37	

### Constipation and LUTS (by subtypes) after 10 years of follow-up

Table 3 shows the associations between constipation and risk of LUTS after 10 years of follow-up. Women who reported taking medication for constipation at only one of the points had an increased risk of urgency (adjusted RR = 1.35; 95% CI 1.04–1.95). The adjusted risk ratio for urgency in women taking constipation medication at both time points was larger (adjusted RR = 1.94; 95% CI 1.15–3.29), but CIs overlapped. The risk of hesitancy was also increased in women taking medication for constipation at only one of the time points (adjusted RR = 1.72; 95% CI 1.04–3.01) and both time points (adjusted RR = 1.78; 95% CI 1.03–4.19). There was no strong evidence of relationships between constipation and any other LUTS subtypes. There was, however, some evidence of a relationship between constipation and risk of any type of LUTS (adjusted RR = 1.54; 95% CI 0.95–2.51) in women reporting medication use for constipation at both time points compared with the reference group, but not for women who reported taking medication at only one time point.

**Table 3.** Associations of Constipation with Lower Urinary Tract Symptoms (by Subtype) at Ten Years of Follow-Up

Outcome	Constipation medication	*N*	*N* cases (%)	Unadjusted RR (95% CI)	Adjusted RR*^a^*(95% CI)
Stress UI					
	None	3,409	762 (22)	Ref	Ref
	Only one time point	225	56 (25)	1.15 (0.84–1.57)	1.17 (0.82–1.65)
	Both time points	87	26 (30)	1.48 (0.92–2.36)	1.37 (0.80–2.31)
Increased daytime frequency					
	None	3,408	483 (14)	Ref	Ref
	Only one time point	227	32 (14)	0.99 (0.68–1.46)	0.90 (0.58–1.40)
	Both time points	87	16 (18)	1.36 (0.79–2.37)	1.46 (0.81–2.64)
Nocturia					
	None	3,408	302 (9)	Ref	Ref
	Only one time point	227	17 (7)	0.83 (0.50–1.38)	0.68 (0.37–1.25)
	Both time points	87	11 (13)	1.49 (0.79–2.83)	1.37 (0.65–2.91)
Urgency UI					
	None	3,404	288 (8)	Ref	Ref
	Only one time point	225	22 (10)	1.17 (0.74–1.85)	0.99 (0.58–1.69)
	Both time points	87	8 (9)	1.10 (0.52–2.29)	1.17 (0.53–2.60)
Urgency					
	None	3,411	584 (17)	Ref	Ref
	Only one time point	225	51 (23)	1.42 (1.03–1.96)	1.35 (1.04–1.95)
	Both time points	87	22 (25)	1.64 (1.00–2.68)	1.94 (1.15–3.29)
Mixed UI					
	None	3,402	274 (8)	Ref	Ref
	Only one time point	225	21 (9)	1.18 (0.74–1.87)	1.07 (0.63–1.84)
	Both time points	87	9 (10)	1.32 (0.65–2.66)	1.29 (0.58–2.87)
Hesitancy					
	None	3,369	155 (5)	Ref	Ref
	Only one time point	222	17 (8)	1.72 (1.02–2.89)	1.72 (1.04–3.01)
	Both time points	86	9 (10)	2.42 (1.19–4.92)	1.78 (1.03–4.19)
Intermittency					
	None	3,403	301 (9)	Ref	Ref
	Only one time point	224	28 (13)	1.47 (0.97–2.22)	1.18 (0.72–1.94)
	Both time points	87	12 (14)	1.65 (0.89–2.89)	1.49 (0.73–2.98)
Any type of LUTS					
	None	3,268	1,310 (40)	Ref	Ref
	Only one time point	214	92 (43)	1.13 (0.85–1.49)	1.07 (0.78–1.46)
	Both time points	82	41 (50)	1.49 (0.96–2.32)	1.54 (0.95–2.51)

^a^ Confounders included for the adjusted models are age, parity, BMI, university degree and social status, physical activity and hysterectomy.

CI, confidence interval; RR, relative risk.

Table 4 shows the findings of the analysis comparing women who reported using medications for constipation at any time point compared with women who reported not using any medication for constipation at either time point. Overall, the findings were similar to the main results. Women who reported using constipation medication had increased risks of urgency (adjusted OR = 1.51; 95% CI 1.10–2.05) and hesitancy (adjusted OR = 1.88; 95% CI 1.16–3.04) compared with women who did not use constipation medication. There was no strong evidence of an association with other LUTS subtypes. In line with the main analysis, there was some evidence of a relationship between constipation and any type of LUTS (adjusted RR = 1.18; 95% CI 0.91–1.55) in women reporting taking medications for constipation at any time point compared with neither.

**Table 4.** Associations of Constipation and Lower Urinary Tract Symptoms (by Subtype) at Ten Years of Follow-Up

Outcome	Constipation medication	*N*	*N* cases (%)	Unadjusted RR (95% CI)	Adjusted RR*^a^*(95% CI)
Stress incontinence					
	None	3,409	76 (22)	Ref	Ref
	Any time point	312	82 (26)	1.24 (0.95–1.61)	1.22 (0.91–1.64)
Frequency					
	None	3,408	483 (14)	Ref	Ref
	Any time point	314	48 (15)	1.09 (0.80–1.51)	1.05 (073–1.50)
Nocturia					
	None	3,408	302 (9)	Ref	Ref
	Any time point	314	28 (9)	1.00 (0.80–1.51)	0.85 (0.53–1.38)
Urgency incontinence					
	None	3,404	288 (8)	Ref	Ref
	Any time point	312	30 (10)	1.15 (0.78–1.71)	1.04 (0.66–1.63)
Urgency					
	None	3,411	584 (17)	Ref	Ref
	Any time point	312	73 (23)	1.48 (1.12–1.95)	1.51 (1.10–2.05)
Mixed incontinence					
	None	3,402	274 (8)	Ref	Ref
	Any time point	312	30 (10)	1.21 (0.78–1.71)	1.13 (0.72–1.28)
Hesitancy					
	None	3,369	155 (5)	Ref	Ref
	Any time point	308	26 (8)	1.91 (1.24–2.94)	1.88 (1.16–3.04)
Intermittency					
	None	3,403	301 (9)	Ref	Ref
	Any time point	311	40 (13)	1.52 (1.07–2.16)	1.26 (0.83–1.91)
Any type of LUTS					
	None	3,268	1,310 (40)	Ref	Ref
	Any time point	296	133 (45)	1.22 (0.95–1.61)	1.18 (0.91–1.55)

^a^ Confounders included for the adjusted models are age, parity, BMI, university degree and social status, physical activity and hysterectomy.

Supplementary Tables S1 and S2 show the results of the complete case analysis, which are similar to the main results.

Supplementary Tables S3 and S4 show the results of the complete case analysis with updated confounders, which were similar to the main analysis (Supplementary Tables S1 and S2).

## Discussion

### Main findings

We found evidence for an increased risk of LUTS after 10 years of follow-up among middle-aged parous women who reported using medication for constipation compared with women who did not use constipation medication. Specifically, women who used medications for constipation had an increased risk of both urgency and hesitancy compared with women who had not used medications for constipation.

### Strengths and limitations

Strengths of our study include its prospective design, large sample size, and use of a validated questionnaire to assess LUTS. We were also able to investigate multiple subtypes of LUTS. We adjusted for potentially important confounders, including hysterectomy and physical activity at baseline. We were also able to adjust for time updated measures of confounders. We used multiple imputation to minimize selection bias due to missing information on potential confounders and to increase statistical efficiency.

Although we had a large sample size, the number of participants taking medication for constipation was relatively small which resulted in wide CIs and, hence, a lack of precision in our results. We categorized constipation as “none, yes at one of the two time points, and yes at both time points” and assumed that women who used medication at both time points have the most severe (chronic) constipation. However, these categories could include women who had mild constipation (*e.g.*, used a laxative once) to severe (*e.g.*, used daily laxatives). Physical activity is a behavior that may change over the 10 years of follow-up, but we were unable to account for changes in physical activity levels after baseline due to a lack of data. Finally, all ALSPAC participants are parous and predominantly white. The results might therefore not be generalizable to nulliparous women or women of other ethnic backgrounds.

### Interpretation of our findings in light of other evidence

A single prospective study (*n* = 234 women recruited while pregnant) has investigated the association between constipation and UI. This study found that chronic constipation (women scoring ≥9 using the Sandwell Incontinence Following Childbirth Risk Assessment Tool risk scale) was associated with an increased risk of stress UI 6 months after childbirth.^22^ In our population of middle-aged women, we did not detect an association between constipation and stress UI.

Kaplan et al. conducted a systematic review of human and animal studies examining the relationship between bowel and bladder function and its implications for managing coexisting constipation and overactive bladder symptoms (OAB), including urgency UI, nocturia, frequency and urgency^8^ and concluded that bowel distension affects bladder activity and that constipation can contribute in the development of OAB symptoms, especially frequency and urgency. This in line with our findings indicating a positive association between constipation and urgency.

Additional support for a causal effect of constipation on LUTS comes from an intervention study examined the effect of alleviating constipation on LUTS, including urgency and frequency. Fifty-two older participants (42 men and 10 women; ages 65–89) with chronic constipation and LUTS self-reported constipation and LUTS by filling in a questionnaire at study enrolment and during monthly visits. The study reported that treating constipation decreased urgency and urinary frequency.^23^

Although, studies mentioned above have reported an increased risk of stress UI and increased daytime frequency in constipated patients,^22,23^ our results suggested a 40% increased risk of increased daytime frequency and stress UI; however, CIs were wide and crossed the null value. This is probably due to limited power as a consequence of the modest number of women who were using constipation medication at baseline.

### Possible mechanisms explaining the association between constipation and LUTS

Constipation left untreated over a long period of time could cause changes in the pelvic floor through several mechanisms, which may result in an increased risk of developing LUTS. Anatomically, the rectum and the bladder are aligned close to each other and therefore share the muscular structure of the pelvic floor. Over time, the cumulative effect of constipation on pelvic floor musculature, may result in high muscle tone, which could cause dysfunctional elimination of urine.^8,9^ Constipation could also cause women to strain while emptying the bowel which could put pressure on the pelvic floor muscles. Continuous straining due to constipation over time could cause weakness in pelvic floor muscles resulting in pelvic organ prolapse, which could increase risk of developing LUTS.^8,9^

The overlapping and convergence of the lower urinary tract and lower bowel innervation can lead to two-way interactions in afferent activity between the two structures. Accordingly, we speculate that the increased afferent activity due to the abnormal presence of feces in the bowel collaterally sensitizes the urinary tract afferents, so the sensory return is disproportionately high for the volume in the bladder. This might also explain the association with hesitancy, since the increased sensation may cause the affected person to visit the toilet when their bladder is underfilled.

## Conclusion

To date, there have been limited prospective studies concerning the association between constipation and LUTS. We found that constipation was prospectively associated with increased risks of urgency and hesitancy among parous middle-aged women. This study potentially highlights that untreated constipation could result in risk of developing LUTS in parous middle-aged women. If further research finds evidence that this association is causal, this implies that women should seek treatment to alleviate constipation to reduce their consequent risk of developing these LUTS.

## Supplementary Material

Supplemental data

Supplemental data

Supplemental data

Supplemental data
