# Supplementary material for: The Association Between Constipation and Lower Urinary Tract Symptoms in Parous Middle-Aged Women: A Prospective Cohort Study
Source: J Womens Health (Larchmt). 2021 Aug 17;30(8):1171–81. doi: 10.1089/jwh.2020.8624 (PMC8403183; doi:10.1089/jwh.2020.8624)
Supplement: Supplemental data [file Supp_TableS2.docx]

**Table S2: Associations of constipation and LUTS (by subtype)**

**At 10 years of follow-up (Complete case analysis)**

| **Outcome** | **Constipation**  **Medication** | **N** | **N cases (%)** | **Unadjusted RR (95% CI)** | **Adjusted RR***  **(95% CI)** |
| --- | --- | --- | --- | --- | --- |
| Stress incontinence |  |  |  |  |  |
|  | None | 2606 | 578 (22) | Ref | Ref |
|  | Any time point | 227 | 54 (24) | 1.07 (0.84, 1.37) | 1.08 (0.84, 1.38) |
| Frequency |  |  |  |  |  |
|  | None | 2606 | 375 (14) | Ref | Ref |
|  | Any time point | 229 | 33 (14) | 1.01 (072, 1.39) | 1.00 (072, 1.39) |
| Nocturia |  |  |  |  |  |
|  | None | 2608 | 224 (9) | Ref | Ref |
|  | Any time point | 229 | 19 (8) | 0.97 (0.62, 1.51) | 0.92 (0.59, 1.43) |
| Urgency incontinence |  |  |  |  |  |
|  | None | 2604 | 218 (8) | Ref | Ref |
|  | Any time point | 227 | 21 (9) | 1.11 (0.72, 1.69) | 1.12 (0.73, 1.69) |
| Urgency |  |  |  |  |  |
|  | None | 2608 | 428 (16) | Ref | Ref |
|  | Any time point | 227 | 52 (23) | 1.40 (1.08, 1.80) | 1.39 (1.08, 1.78) |
| Mixed  incontinence |  |  |  |  |  |
|  | None | 2602 | 205 (8) | Ref | Ref |
|  | Any time point | 227 | 19 (8) | 1.06 (0.68, 1.67) | 1.06 (0.68, 1.66) |
| Any type of LUTS |  |  |  |  |  |
|  | None | 2501 | 995 (40) | Ref | Ref |
|  | Any time point | 214 | 93 (44) | 1.09 (0.93, 1.28) | 1.08 (0.92, 1.27) |
| Hesitancy |  |  |  |  |  |
|  | None | 2577 | 125 (5) | Ref | Ref |
|  | Any time point | 224 | 19 (9) | 1.83 (1.23, 2.73) | 1.75 (1.10, 2.78) |
| Intermittency |  |  |  |  |  |
|  | None | 2603 | 233 (9) | Ref | Ref |
|  | Any time point | 226 | 24 (11) | 1.45 (1.07, 1.98) | 1.16 (0.78, 1.72) |
| **Footnote**  ***** Confounders included for the adjusted models are age, parity, BMI, university degree and social status, physical activity and hysterectomy. | | | | | |
