# Supplementary material for: The Association Between Constipation and Lower Urinary Tract Symptoms in Parous Middle-Aged Women: A Prospective Cohort Study
Source: J Womens Health (Larchmt). 2021 Aug 17;30(8):1171–81. doi: 10.1089/jwh.2020.8624 (PMC8403183; doi:10.1089/jwh.2020.8624)
Supplement: Supplemental data [file Supp_TableS3.docx]

**Table S3: Sensitivity analysis of Associations of constipation and LUTS (by subtype)**

**At 10 years of follow-up with updated confounders(Complete case analysis)**

| **Outcome** | **Constipation Medication** | **N** | **N cases (%)** | **Unadjusted RR (95% CI)** | **Adjusted RR* (95% CI)** |
| --- | --- | --- | --- | --- | --- |
| Stress incontinence |  |  |  |  |  |
|  | None | 2606 | 578 (22) | Ref | Ref |
|  | Either time points | 165 | 40 (24) | 1.09 (0.83, 1.44) | 1.03 (0.71, 1.40) |
|  | Both time points | 62 | 14 (23) | 1.02 (0.64, 1.62) | 1.00 (0.38, 1.41) |
| Frequency |  |  |  |  |  |
|  | None | 2606 | 375 (14) | Ref | Ref |
|  | Either time points | 167 | 23 (14) | 0.95 (0.64, 1.41) | 0.92 (0.59, 1.46) |
|  | Both time points | 62 | 10 (15) | 1.05 (0.57, 1.92) | 1.20 (0.67, 2.21) |
| Nocturia |  |  |  |  |  |
|  | None | 2608 | 224 (9) | Ref | Ref |
|  | Either time points | 167 | 12 (7) | 0.84 (0.48, 1.46) | 0.50 (0.23, 1.06) |
|  | Both time points | 62 | 7 (11) | 1.31 (0.65, 2.67) | 1.46 (0.69, 3.15) |
| Urgency incontinence |  |  |  |  |  |
|  | None | 2604 | 218 (8) | Ref | Ref |
|  | Either time points | 165 | 15 (9) | 1.09 (0.66, 1.79) | 1.00 (0.54, 1.70) |
|  | Both time points | 62 | 6 (10) | 1.16 (0.53, 2.50) | 1.11 (0.39, 2.62) |
| Urgency |  |  |  |  |  |
|  | None | 2608 | 428 (16) | Ref | Ref |
|  | Either time points | 165 | 37 (22) | 1.37 (1.01, 1.84) | 1.35 (1.00, 1.89) |
|  | Both time points | 62 | 15 (24) | 1.47 (0.94, 2.31) | 1.35 (0.79, 2.33) |
| Mixed  incontinence |  |  |  |  |  |
|  | None | 2602 | 205 (8) | Ref | Ref |
|  | Either time points | 165 | 14 (9) | 1.08 (0.64, 1.81) | 1.06 (0.47, 1.68) |
|  | Both time points | 62 | 5 (8) | 1.02 (0.44, 2.40) | 1.10 (0.43, 2.83) |
| Any type of LUTS |  |  |  |  |  |
|  | None | 2501 | 995 (40) | Ref | Ref |
|  | Either time points | 157 | 66 (42) | 1.06 (0.87, 1.28) | 1.03 (0.84, 1.26) |
|  | Both time points | 55 | 27 (47) | 1.19 (0.90, 1.57) | 1.12 (0.90, 1.49) |
| Hesitancy |  |  |  |  |  |
|  | None | 2577 | 125 (5) | Ref | Ref |
|  | Either time points | 162 | 14 (9) | 1.66 (1.03, 2.70) | 1.65 (1.04, 2.96) |
|  | Both time points | 62 | 5 (8) | 2.27 (1.20, 4.30) | 1.86 (0.85, 4.14) |
| Intermittency |  |  |  |  |  |
|  | None | 2603 | 233 (9) | Ref | Ref |
|  | Either time points | 164 | 18 (11) | 1.41 (0.98, 2.03) | 1.17 (0.76, 1.80) |
|  | Both time points | 62 | 6 (10) | 1.56 (0.91, 2.67) | 1.15 (0.53, 2.33) |
| **Footnote**  ***** Confounders included for the adjusted models are :  Age and BMI (measure at 10 years follow up from baseline)  Parity and hysterectomy (measured at 8 years of follow up from baseline)  Physical activity measured at baseline  University degree, social status measured before baseline | | | | | |
